# Toxicity after prolonged (more than four weeks) administration of intravenous colistin

**DOI:** 10.1186/1471-2334-5-1

**Published:** 2005-01-10

**Authors:** Matthew E Falagas, Michael Rizos, Ioannis A Bliziotis, Kostas Rellos, Sofia K Kasiakou, Argyris Michalopoulos

**Affiliations:** 1 Alfa Institute of Biomedical Sciences, Athens, Greece; 2 Tufts University School of Medicine, Boston, Massachusetts, USA; 3 Alfa HealthCare, Athens, Greece; 4 Intensive Care Unit, "Henry Dunant" Hospital, Athens, Greece

## Abstract

**Background:**

The intravenous use of polymyxins has been considered to be associated with considerable nephrotoxicity and neurotoxicity. For this reason, the systemic administration of polymyxins had been abandoned for about 20 years in most areas of the world. However, the problem of infections due to multidrug-resistant (MDR) Gram-negative bacteria such us *Pseudomonas aeruginosa *and *Acinetobacter baumanniii *has led to the re-use of polymyxins. Our objective was to study the toxicity of prolonged intravenous administration of colistin (polymyxin E).

**Methods:**

An observational study of a retrospective cohort at "Henry Dunant" Hospital, a 450-bed tertiary care center in Athens, Greece, was undertaken.

Patients who received intravenous colistin for more than 4 weeks for the treatment of multidrug resistant Gram-negative infections were included in the study. Serum creatinine, blood urea, liver function tests, symptoms and signs of neurotoxicity were the main outcomes studied.

**Results:**

We analyzed data for 19 courses of prolonged intravenous colistin [mean duration of administration (± SD) 43.4 (± 14.6) days, mean daily dosage (± SD) 4.4 (± 2.1) million IU, mean cumulative dosage (± SD) 190.4 (± 91.0) million IU] in 17 patients. The median creatinine value increased by 0.25 mg/dl during the treatment compared to the baseline (p < 0.001) but returned close to the baseline at the end of treatment (higher by 0.1 mg/dl, p = 0.67). No apnea or other evidence of neuromuscular blockade was noted in any of these patients who received prolonged treatment with colistin.

**Conclusions:**

No serious toxicity was observed in this group of patients who received prolonged intravenous colistin. Colistin should be considered as a therapeutic option in patients with infections due to multidrug resistant Gram-negative bacteria.

## Background

The worldwide spread of the problem of infections due to Gram-negative bacteria such us *Pseudomonas aeruginosa *and *Acinetobacter baumannii *resistant to most classes of antimicrobial agents has made the medical community to rethink of colistin, an old, basically abandoned for the last two decades antibiotic. Intravenous colistin (polymyxin E) has been used in the industrialized countries during the last years mainly in patients with cystic fibrosis infected with *Pseudomonas aeruginosa *resistant to other available antimicrobial classes [1]. Excluding this population, the agent has been infrequently used during the last two decades because of major concerns about nephrotoxicity and neurotoxicity [2].

Several reports during the early years of use of the medication, mainly in the decade 1960 to 1969 left the medical community with the impression that the medication is very toxic [3,4]. However, the recent experience of several clinicians worldwide with the use of the medication has not verified the old reports about the serious or common toxicity of colistin [5-8]. We analysed data of a group of patients who received colistin for more than four weeks to investigate further the concerns about the toxicity of the medication.

## Methods

### Design of the study-patient selection

Patients who received intravenous colistin from 1/October/2000 to 31/January/2004 at "Henry Dunant" Hospital, a 450-bed tertiary care center in Athens, Greece, were identified by the pharmacy electronic databases. All patients who were found to have received intravenous colistin for more than four weeks continuously, or in two courses of colistin but with no more than seven days interruption between the courses were included in this study. Administration of intravenous colistin in two courses for more than four weeks in each course were studied as two different units in the analysis of toxicity, if there was more than one month colistin-free period between the two courses. One milligram of the colistin formulations used is approximately equal to 12.500 IU (Colomycin, Forest Laboratories^®^, Kent, UK or Colistin, Norma^®^, Athens, Greece). The study was approved by the Institutional Review Board (IRB) of the hospital.

### Definitions of infections

Diagnosis of pneumonia required two or more serial chest radiographs with at least one of the following: new or progressive and persistent infiltrate, consolidation, cavitation, or pleural effusion. In addition, patients must have had fever >38°C with no other recognized cause, or abnormal white blood cell count [leukopenia (< 4000 WBC/mm^3^) or leukocytosis (= 12.000 WBC/mm^3^)], and at least two of the following: new onset of purulent sputum or change in character of sputum, increased respiratory secretions or increased suctioning requirements, new onset or worsening of cough or dyspnea or tachypnea, rales or bronchial breath sounds, or worsening gas exchange [9].

Bacteremia required either growth of a recognized pathogen from one or more blood specimen cultures or at least one of the following signs or symptoms: fever (>38°C), chills, or hypotension *and *at least one of the following: a) common skin contaminant (e.g., diphtheroids, *Bacillus *sp., *Propionibacterium *sp., coagulasenegative staphylococci, or micrococci) grown from two or more blood cultures drawn on separate occasions or b) common skin contaminant (e.g., diphtheroids, *Bacillus *sp., *Propionibactαium *sp., coagulase-negative staphylococci, or micrococci) grown from at least one blood culture from a patient with an intravascular line and physician- instituted antimicrobial therapy [9].

Infections at other body sites or fluids, such as urinary tract infections and surgical site infections were defined based on guidelines from the Centers for Disease Control and Prevention [9]. Clinical specimens of all body sites were considered when defining the etiologic microbiologic agent of the infection.

### Data collection-entry

Data for several variables including demographic and clinical information as well as results of laboratory and imaging tests were collected and entered in a computer database. All available results of renal function tests (serum creatinine, urea, creatinine clearance, urinalysis), liver function tests (SGPT, SGOT, alkaline phosphatase, γ-GT, bilirubin), creatine phosphokinase (CPK) during the course of colistin treatment and at hospital discharge were collected.

### Data analysis

Creatinine values at the beginning of colistin treatment were compared with the maximum value of creatinine during therapy as well as with the creatinine value at the end of treatment using a non-parametric test (Wilcoxon). The data analysis was performed using SPSS and S-plus software.

## Results

### Study population

During the period from 1/October/2000 to 31/January/2004, 152 patients received intravenous colistin. Data about the efficacy of the medication were analysed and reported elsewhere [5]. In this report, we present detailed data regarding the toxicity of colistin in a subgroup of 17 patients who received more than four weeks of intravenous colistin. Table 1 describes various characteristics of this group of patients including demographic and clinical data, such as comorbidity, site of infection, and responsible pathogens. Two patients received two prolonged courses of intravenous colistin each with more than one month colistin-free period between the courses. Subsequently, there were 19 courses of prolonged colistin for the analysis of toxicity. Among these 19 courses, the administration of intravenous colistin was without interruption in 15 courses, while there was interruption of colistin administration in 2 courses for two days, interruption in 1 course for 3 days, and interruption in 1 course for 4 days. The cause for the interruption of colistin in these cases was the attending physicians' attempts to obtain cultures without the confounding influence of antimicrobial treatment in patients with puzzling continuing symptoms of infection. The mean duration of colistin administration (± SD), for each course of colistin therapy was 43,4 days (± 14,6 days). The mean daily dosage ± SD for each course was 4.4 million IU (352 mg) ± 2.1 million IU (168 mg) and the mean cumulative dosage of colistin ± SD for each course was 190.4 million IU (15.232 mg) ± 91.0 million IU (7.280 mg). The mean daily dosage for a patient with body weight of 70 kg was 62.857 IU/kg (5 mg/kg). For patients with impaired renal function, dosage adjustments were done mainly after consulting the ICU director or the Infectious Disease specialists of the hospital, based on the following protocol: if serum creatinine level was 1.3–1.5 mg/dl, 1.6–2.5 mg/dl or = 2.6 mg/dl, the dosage of colistin administered was 2 million IU (160 mg) every 12 hours, 24 hours, or 36 hours, respectively. Patients who were on dialysis treatment received 1 million IU (80 mg) of colistin after dialysis. Colistin was given for long duration in patients mainly in the ICU setting, because of persistence of infections due to Gram-negative bacteria sensitive only to colistin or bacteria sensitive also to antibiotics of other classes that were previously administered (based on in-vitro susceptibility test results) but failed. Subsequently, no other therapeutic strategy was considered better than the continuation of colistin (as combination therapy or monotherapy) because of failure of previous antibiotic regimens and the lack of other therapeutic options.

**Table 1:** Demographic and clinical features of patients managed with prolonged intravenous colistin for infections caused by multidrug-resistant Gram-negative bacteria.

**Characteristics of patients**	**Median (range) n (%)**
**Demographic**	
Age, years [median (range)]	51 (18 – 79)
Sex, male	12/17 (70%)
**APACHE II score**	
On admission to ICU [median (range)]	14 (7 – 35)
On 1^st ^day of colistin treatment [median (range)]	14 (6 – 22)
**Comorbidity**	
Malignancy	2/17 (11%)
Hemodialysis	2/17 (11%)
Urogenital disorders	3/17 (18%)
Heart dysfunction	5/17 (29%)
Diabetes mellitus	5/17 (29%)
Lung dysfunction	5/17 (29%)
Liver failure	1/17 (6%)
Hematological disorders	3/17 (18%)
Neurological disorders	8/17 (47%)
Neuropathy/myopathy	4/17 (23%)
Trauma	7/17 (41%)
**Transfusion**	15/17 (88%)
**Prior hospitalization**	12/17 (70%)
**Prior surgery**	14/17 (82%)
Elective	9/14 (64%)
Emergency	5/14 (36%)
**Prosthetic material**	11/17 (64%)
**Catheters/Invasive devices**	
Tracheostomy	14/17 (82%)
CSF drainage	5/17 (29%)
**Prior medications**	
Prior antibiotic use	17/17 (100%)
Prior antifungal use	8/17 (47%)
Anti-tumor treatment	0/17 (0%)
Cortisone treatment	9/17 (53%)
**Duration of hospitalization (days) **[median (range)]	152 (29 – 591)
**Duration of stay in ICU (days) **[median (range)]	70 (22 – 134)
Site of infection*	
Pneumonia	13/19 (68%)
Bacteremia	1/19 (5%)
Urinary tract infection	2/19 (11%)
Meningitis	2/19 (11%)
Surgical site infection	1/19 (5%)
**Pathogens†**	
*Pseudomonas aeruginosa*	12/20 (60%)
*Acinetobacter baumannii*	5/20 (25%)
*Klebsiella pneumoniae*	2/20 (10%)
*Enterobacter cloacae*	1/20 (5%)
**Mortality**	7/17 (41%)

* The analysis of the site of infection was based on 19 episodes of infection in 17 patients.

**† **The analysis of the responsible pathogens was based on 20 isolates from 19 episodes of infection in 17 patients [two pathogens were isolated in one episode of pneumonia (*Klebsiella pneumoniae + Acinetobacter baumannii *mixed infection)].

The analysis of the responsible pathogens was based on 20 isolates from 19 episodes of infection in 17 patients (two pathogens were isolated in one episode). In 2 episodes of infection, both caused by *Pseudomonas aeruginosa *strains, the minimum inhibitory concentrations (MICs) were not available. In the remaining 10 *Pseudomonas aeruginosa *isolates, the MICs to colistin ranged from = 0.5 mg/l to 2 mg/l. In vitro susceptibility testing for the 5 isolated *Acinetobacter baumannii *strains revealed MICs to colistin = 0.5 mg/l, for the 2 *Klebsiella pneumoniae *strains MICs were 0.5 mg/l and 2 mg/l, and for the *Enterobacter cloacae *strain MIC was 1 mg/l.

The all-cause in-hospital mortality was 41,2% (7 out of 17 patients). Clinical cure and improvement, defined as resolution and partial resolution, respectively, of presenting symptoms and signs of the infection by the end of colistin treatment was observed in 14 of the 19 episodes of infection (73.7%) [cure 10/19 episodes (52.6%), improvement 4/19 episodes (21.1%)]. Unresponsiveness, defined as persistence or worsening of presenting symptoms and/or signs of the infection during intravenous colistin administration, was observed in 5 of the 19 episodes of infection (26.3%).

All patients had been admitted to the ICU at some time during their hospitalisation. In 12 courses out of the 19 analyzed the whole colistin therapy was administered in the ICU, in 1 course it was administered in a hospital ward, and in 6 courses it was given both in the ICU and a hospital ward.

### Renal toxicity

**Figure 1:**
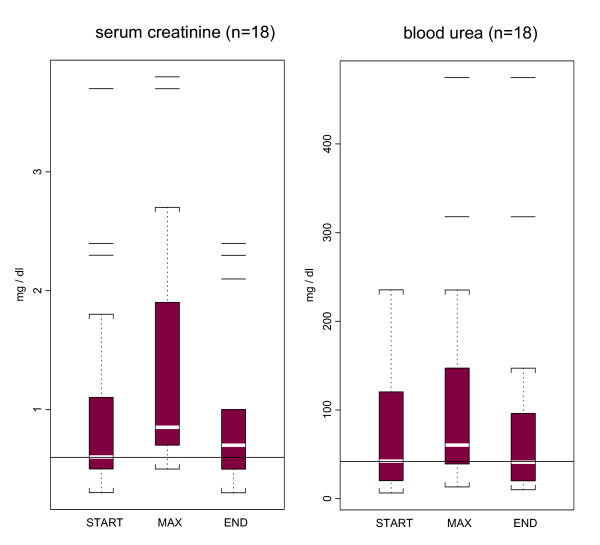
The distribution of serum creatinine and blood urea levels on the 1^st ^day of colistin treatment (start), at the peak value (max), and the end of colistin treatment (end) in all studied courses of prolonged treatment with colistin. (The horizontal line within the boxes represents the median creatinine or blood urea baseline value at the 1^st ^day of colistin treatment. "Shaded" boxes in the figures represent the distribution of the laboratory values contained between the 25^th ^and 75^th ^percentiles (the interquartile range). Dotted lines (whisker lines) extend from the box, down and up, to the minimum and maximum values of the distributions that are not outliers (i.e. that are within 1.5 times the interquartile range); these values of the distribution are shown as "brackets". Any value, which is an outlier, is drawn as a "horizontal line").

Figure 1 depicts the box plots of serum creatinine and blood urea values of patients on the first day of colistin, the maximum values during the course of administration of colistin, and the values at the end of the treatment with colistin (one patient, who was on hemodialysis treatment prior to and during the colistin course, was removed from the analysis of the comparisons of the creatinine and urea values). The median value of baseline creatinine at the beginning of colistin therapy for the 18 prolonged courses of colistin was 0,6 mg/dl. Compared to this value, there was a slight increase of the median of values of creatinine at the end of the colistin course by 0,1 mg/dl, without statistical significance (p = 0,67). The median of the maximum values of creatinine observed was 0,85 mg/dl and was found to be statistically different from both the median of start (p < 0,001) and end of treatment values (p < 0,001). The maximum absolute increase of creatinine, compared to baseline, observed in an episode was 1,4 mg/dl. Only one patient had an increase of more than 50% of the baseline creatinine level to a value higher than 1.3 mg/dl at the end of colistin treatment. For the subgroup of 12 patients with 14 courses of prolonged colistin administration, with normal serum creatinine at the initiation of treatment, the median serum creatinine values at baseline and at the end of colistin treatment were 0,60 mg/dl and 0,55 mg/dl, respectively. In addition, the median maximum value of serum creatinine during the course of colistin treatment was 0,80 mg/dl among this group of patients.

At the initiation of colistin administration the median value of baseline blood urea for the 18 courses was 42 mg/dl. During the 18 prolonged courses of colistin administration there was a slight increase in blood urea values; the median maximum value was 60 mg/dl. However, at the completion of colistin therapy median blood urea value returned to 41 mg/dl.

During the 19 courses of colistin administration, other potentially nephrotoxic drugs were given. Specifically, aminoglycosides were co-administered in 12 courses [median duration of co-administration (± SD) in these 12 courses 12,5 (± 16,8) days], teicoplanin in 10 [median duration of co-administration 16,0 (± 9,0) days], vancomycin in 8 [median duration of co-administration 23,0 (± 11,2) days], amphotericin in 4 [median duration of co-administration 8,0 (± 9,0) days].

Four patients had history of chronic renal dysfunction, with only one of them receiving hemodialysis treatment prior to admission. This patient continued the hemodialysis during his treatment with colistin and died due to sepsis. For the other 3 patients the baseline, the maximum, and the end-of-therapy creatinine values (mg/dl), were 1,8/2,1/2,1 for the first patient, 3,7/3,7/2,4 for the second patient and 2,4/3,8/2,3 for the third patient.

Only one patient had acute renal failure prior to colistin use, which was due to severe haemorrhage during surgery. This patient received 13 courses of hemodialysis. One day after the last course, colistin therapy had to be initiated for the management of *Acinetobacter baumannii *pneumonia, sensitive only to colistin and gentamicin. Apart from a slight elevation of serum creatinine (from 2,3 mg/dl to 2,7 mg/dl) on the 5^th ^day of treatment no other sign of nephrotoxicity was observed during a 34-day administration of colistin (the 9^th ^day creatinine returned to 0,7 mg/dl).

### Neuromuscular toxicity

No apnea or other evidence of neuromuscular blockade was noted in any of these patients who received prolonged treatment with colistin. Four patients were clinically diagnosed to have polyneuropathy and/or myopathy before or during colistin treatment. In the first patient the polyneuropathy symptoms appeared while she was on her 25^th ^day of colistin treatment and became worse on the 27^th ^day. From then on, and although colistin was continued for 11 more days, the symptoms gradually subsided. No confirmatory electromyographic testing was performed. The second patient was transferred from another ICU where he was diagnosed as having myopathy due to sepsis. Fifteen days after his admission, and while his myopathy was improving, therapy with colistin was initiated; the treatment did not affect the gradual improvement. An electromyography performed on his 21^st ^day in our ICU, showed mild axial sensory-motor polyneuropathy and myopathy. He received colistin for a total of 52 days and made full recovery from his neuropathy/myopathy during the course of colistin administration. The third patient developed ICU polyneuropathy one week prior to colistin administration and, again, recovered gradually from it while he was on colistin treatment. The fourth patient was transferred from another ICU, where he had developed ICU polyneuropathy. Only this patient had moderate worsening of the neuropathy while he was receiving colistin. Despite that, he received colistin for a total of 35 days for a respiratory infection with persisting *Pseudomonas aeruginosa *sensitive only to colistin. His neuropathy improved gradually after the end of colistin treatment. Thus, our assessment regarding these 4 patients who experienced polyneuropathy and/or myopathy is that colistin therapy was probably associated with the development of neurotoxicity only in one of the 4 patients.

### Liver and biliary tree toxicity

**Figure 2:**
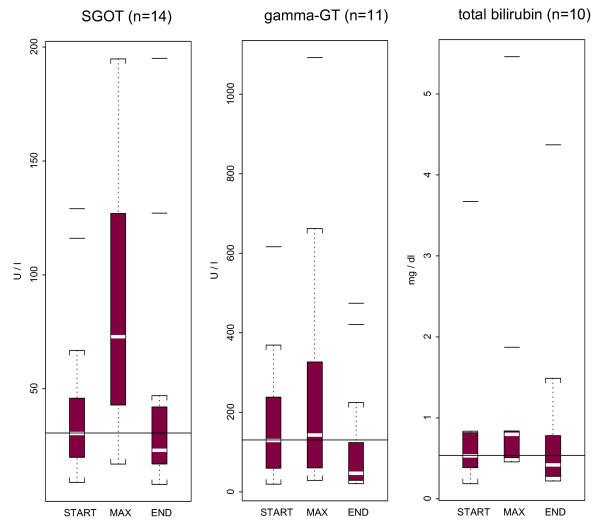
The distribution of liver function tests [SGOT (AST = aspartate aminotransferase), γ-GT, and total bilirubin] on the 1^st ^day of colistin treatment (start), at the peak value (max), and at the end of colistin treatment (end) in studied courses of prolonged treatment with colistin. (The horizontal line within the boxes represents the median creatinine or blood urea baseline value at the 1^st ^day of colistin treatment. "Shaded" boxes in the figures represent the distribution of the laboratory values contained between the 25^th ^and 75^th ^percentiles (the interquartile range). Dotted lines (whisker lines) extend from the box, down and up, to the minimum and maximum values of the distributions that are not outliers (i.e. that are within 1.5 times the interquartile range); these values of the distribution are shown as "brackets". Any value, which is an outlier, is drawn as a "horizontal line").

Data for the possible hepatobiliary toxicity of colistin were collected. Both laboratory and clinical findings were taken into consideration. In subgroup analyses of patients for whom data were available, no substantial changes on liver function tests was found, as described in Figure 2 (data for SGPT, alkaline phosphatase, direct and indirect bilirubin are not shown). Three of our patients were found to have increased levels of hepatic and cholestatic enzymes during colistin administration. One of them was found to have acute cholecystitis, the second had severe inflammatory systemic reaction (SIRS), and the third had transient elevation of transaminases while he was receiving anti-epileptic medications together with colistin.

## Conclusions

The main finding of the study is that no significant deterioration of renal function was found in patients with baseline normal serum creatinine value during prolonged administration of intravenous colistin. In addition, in the group of patients with pre-existing chronic renal dysfunction (not on hemodialysis) the administration of the medication for a long duration did not influence further their renal function. The observed slight increase in the serum creatinine values at the end of treatment (0,1 mg/dl increase in the median creatinine values from the start to end of colistin administration) cannot be attributed only to the prolonged colistin administration as other factors such as concomitantly administered potential nephrotoxic agents, and sepsis per ce may have had an effect. In addition, apnea or other evidence of neuromuscular blockade was not observed in any of the 17 patients who received prolonged treatment with colistin.

Early experience with colistin revealed a high incidence of toxicity, mainly nephrotoxicity. In 1969, Ryan et al. reported the fatal case of a previously healthy 4-year-old child with persistent fever after appendectomy who died due to cardiac arrest [10]. The child had received colistin intramuscularly due to the growth of *Pseudomonas aeruginosa *and *E. coli *strains from the excised specimen. This patient developed acute tubular necrosis (confirmed by autopsy) and apnea requiring intubation and mechanical ventilation. However, in this case the dosage of colistin used was 30 mg/kg of body weight every six hours (120 mg/kg/day) whereas the recommended dose is 2,5 – 5 mg/kg/day. One year later, several reports showed high renal adverse reaction rates after colistin administration [2,4,11]. In many of them the dosage used was also higher compared to the recommended, i.e. 6,3 mg/kg/day in one report, and 26 mega units in another administered as 10 mega units by intramuscular injection, 10 mega units by intravenous injection and 6 mega units by inhalation (no details are provided in the report about the ratio of IU of colistin per mg). In addition, dosage adjustments in patients with renal dysfunction were not always followed, as well as there were also other factors which perhaps contributed to toxicity, such as the co-administration of other medications with potential toxicity, including anesthetic drugs and muscle relaxants.

However, recent data from published reports do not corroborate this finding [14]. Notably, one study conducted in 35 patients with ventilator-associated pneumonia due to multidrug-resistant *Acinetobacter baumannii*, who received either intravenous colistin (21 patients) or imipenem (14 patients), showed an almost double increase in nephrotoxicity rates among the patients in the imipenem group compared to colistin group [12]. In addition, Stein et al. recently reported their experience from 3 patients with orthopedic infections who received colistin for 3 – 6 months without developing any adverse effect requiring discontinuation of the treatment [13].

The conclusion in the majority of the published studies during 1960 – 1970 was that colistin must be used in serious infections where other antimicrobial agents have failed, always with close monitor of renal function, and with precaution in the co-administration of other possible nephrotoxic or neutotoxic agents [2,4]. Subsequently, the considerable difference regarding reporting toxicity of colistin between old and recent studies deserves an explanation. First, the formulations of currently used colistin may be more purified compared with the old ones. The experience that was accumulated from the use of vancomycin may support this possibility. It is known that the concerns about serious and/or common nephrotoxicity of vancomycin overwhelmed the older literature. Second, fluid supplementation and supportive treatment of severely ill patients has been improved nowadays. Third, higher doses of colistin were administered to patients during the first years of its use.

Our study is not without limitations. It is a retrospective study with the inherited problems related to this study design, including difficulties in the determination of colistin associated sensory neurotoxicity. In addition, the number of studied patients is relatively small and there is no control group. However, our study offers insight related to interesting experience from a group of patients who received prolonged treatment with colistin for various infections, mainly as a salvage treatment of persisting and serious infections.

In conclusion, intravenous colistin seems to be a safe therapeutic intervention for serious infections due to multidrug-resistant Gram-negative bacteria even when it is administered for a prolonged duration (more than four weeks). More studies will be needed to further clarify the correct use of this old antibiotic in the 21^st ^century.

## Competing interests

The author(s) declare that they have no competing interests.

## Authors' contribution

MEF and AM conceived the idea; MR, IAB, KR, and SKK collected the data; all authors contributed in the writing and preparation of the manuscript.

## Pre-publication history

The pre-publication history for this paper can be accessed here:


